# Impact of comorbidity assessment methods to predict non-cancer mortality risk in cancer patients: a retrospective observational study using the National Health Insurance Service claims-based data in Korea

**DOI:** 10.1186/s12874-021-01257-2

**Published:** 2021-04-09

**Authors:** Sanghee Lee, Yoon Jung Chang, Hyunsoon Cho

**Affiliations:** 1 Department of Cancer Control and Population Health, Graduate School of Cancer Science and Policy, National Cancer Center, 323 Ilsan-ro, Ilsandong-gu, Goyang, 10408 Republic of Korea; 2 National Cancer Survivorship Center, National Cancer Control Institute, National Cancer Center, Goyang, Republic of Korea

**Keywords:** Comorbidity, Cancer, Claims data, Charlson comorbidity index, Non-cancer, Mortality, Prognosis prediction

## Abstract

**Background:**

Cancer patients’ prognoses are complicated by comorbidities. Prognostic prediction models with inappropriate comorbidity adjustments yield biased survival estimates. However, an appropriate claims-based comorbidity risk assessment method remains unclear. This study aimed to compare methods used to capture comorbidities from claims data and predict non-cancer mortality risks among cancer patients.

**Methods:**

Data were obtained from the National Health Insurance Service-National Sample Cohort database in Korea; 2979 cancer patients diagnosed in 2006 were considered. Claims-based Charlson Comorbidity Index was evaluated according to the various assessment methods: different periods in washout window, lookback, and claim types. The prevalence of comorbidities and associated non-cancer mortality risks were compared. The Cox proportional hazards models considering left-truncation were used to estimate the non-cancer mortality risks.

**Results:**

The prevalence of peptic ulcer, the most common comorbidity, ranged from 1.5 to 31.0%, and the proportion of patients with ≥1 comorbidity ranged from 4.5 to 58.4%, depending on the assessment methods. Outpatient claims captured 96.9% of patients with chronic obstructive pulmonary disease; however, they captured only 65.2% of patients with myocardial infarction. The different assessment methods affected non-cancer mortality risks; for example, the hazard ratios for patients with moderate comorbidity (CCI 3–4) varied from 1.0 (95% CI: 0.6–1.6) to 5.0 (95% CI: 2.7–9.3). Inpatient claims resulted in relatively higher estimates reflective of disease severity.

**Conclusions:**

The prevalence of comorbidities and associated non-cancer mortality risks varied considerably by the assessment methods. Researchers should understand the complexity of comorbidity assessments in claims-based risk assessment and select an optimal approach.

**Supplementary Information:**

The online version contains supplementary material available at 10.1186/s12874-021-01257-2.

## Background

Advances in cancer diagnosis and treatment have extended the life expectancy in cancer patients and increased cancer survivors. However, these survivors now face an increased risk of non-cancer-related death [1] due to comorbidities and complications associated with cancer treatments [2, 3].

The prognosis prediction tools that incorporate comorbidity may be useful in facilitating clinical decisions and understanding how the patient’s comorbidity affects survival outcomes further [3, 4]. Therefore, the assessment of comorbid conditions and their impact on non-cancer mortality risks are critical to cancer prognostication. Further, predictive models not adjusted for comorbidities may yield biased survival estimates. Because many cancer registries do not record patients’ comorbid conditions before a cancer diagnosis, administrative claims data are often considered data sources for comorbid conditions [5]. Indeed, comorbid conditions assessment from the claims data can also be used for health service planning or population health monitoring. Population-based claims data can primarily provide a more representative and comprehensive picture of the health status of cancer patients.

The Charlson Comorbidity Index (CCI) is a widely used metric and developed to account for 19 comorbid conditions in medical records [6]. Several diagnostic coding algorithms, which use the International Statistical Classification of Diseases and Related Health Problems (ICD) codes, have been developed to extract information on CCI conditions from claims-based health care data. In 1992, Deyo et al. adapted the CCI to the International Classification of Diseases, Ninth Revision, Clinical Modification (ICD-9-CM), using inpatient claims only [7]. Subsequently, the diagnostic codes were updated in 2002, 2004, and 2005 following the introduction of the Tenth Revision of the International Statistical Classification of Diseases and Related Health Problems (ICD-10), as proposed by Halfon et al., Sundararajan et al., and Quan et al., respectively [8–10]. Of these, the diagnostic coding system devised by Quan et al. demonstrated a superior median discriminative ability to predict the overall mortality risk [11]. The ongoing shift toward delivering health care services in outpatient settings has led to increases in the prevalence of comorbidities observed therein. In 2000, Klabunde et al. developed a comorbidity index that accounted for comorbid conditions in outpatient claims and simultaneously used the rule-out algorithm to prevent the up-coding of these claims [12].

Meanwhile, studies also examined the impact of the comorbidity ascertainment period [5, 13–18] on survival estimates [12, 19–23]. Preen et al. evaluated the effects of different lookback periods on post-discharge mortality and readmission risk estimates [20]. Kim et al. compared different lookback periods when predicting in-hospital mortality for patients who underwent percutaneous coronary intervention. However, they recommended further study to determine the adequate ascertainment periods for patients with other diseases [22].

Most studies measured comorbid conditions based on practical considerations such as convenience, experience, and data availability rather than an empirical evaluation of comorbidity risks [19, 20]. Further, no previous studies conducted a systematic assessment of the impact of different comorbidity assessment methods on the estimates of non-cancer mortality risks among cancer survivors. This study demonstrated the potential issues of ascertainment periods and claim types in capturing comorbid conditions using the administrative claims data. Moreover, we aimed to evaluate the effect of different comorbidity assessment methods on the prevalence of comorbidities and their associated non-cancer mortality risk.

## Methods

### Data sources

We used de-identified secondary data of a population-based sample cohort of 1,000,000 participants established by the National Health Insurance Service (NHIS) in Korea [24]. A representative sample cohort, comprising 2% of the total eligible Korean population in 2006, was selected randomly and followed until 2015 (10 years). The database contains data on the demographics, medical aid, medical bills, medical treatments, and prescriptions, which were retrospectively collected from 2002 until 2015. The database is linked to the mortality and cause of death statistics provided by the Korea National Statistical Office, follow-up through Dec 31, 2015.

### Study population

We identified cancer patients diagnosed in 2006 using ICD-10 codes corresponding to malignant neoplasms (C00–C97). Patients with prior cancer history were excluded. The final study cohort includes patients who had been continuously enrolled in health insurance for at least 3 years (2002–2005) before cancer diagnosis to ensure comparability in comorbid conditions measurements across different lookback periods (1, 2, and 3 years).

### Measurement

We identified the Charlson comorbid conditions using the ICD-10 diagnostic coding system proposed by Quan et al. [8] and applied the rule-out algorithm developed by Klabunde et al. [12]. Although Klabunde et al. used a 1-month washout window period to prevent the up-coding of outpatient claims [12], different washout window periods of 0, 30, and 90 days were additionally evaluated and compared in this study. We further considered various lookback periods (1, 2, and 3 years) and claim types (inpatient claims only, outpatient claims only, and either inpatient or outpatient claims) [see Additional file 1]. A total of 27 different comorbidity assessment methods, consisting of a combination of washout window periods, lookback period, and claims types, were compared to estimate comorbidity prevalence and its impact on non-cancer mortality.

In this study, the cancer-related comorbidities in CCI were not considered: any malignancy including lymphoma and leukaemia except malignant neoplasm of the skin (C00–C26, C30–C34, C37–C41, C43, C45–C58, C60–C76, C81–C85, C88, and C90–C97) and solid metastatic tumour (C77–C80). Besides, dementia (F00-F03, F05.1, G30, G31.1) and AIDS/HIV (B20, B22, B24) were masked in the database due to NHIS data confidentiality policy.

### Statistical analysis

We estimated non-cancer mortality using the Cox proportional hazards model considering left truncated and right-censored data. The hazard ratios (HR) and 95% confidence intervals (95% CIs) of non-cancer mortality were estimated. Although cancer survival studies typically use the time since cancer diagnosis, this study used patient age as the timescale to describe the impacts of comorbidities on the non-cancer mortality risk considering a left truncated feature of the data. The left truncation occurs because patients entered the study at the time of cancer diagnosis, rather than at the start of the timeline (i.e., birth) [2].

The CCI was calculated by summing the weights of individual comorbidities derived by Charlson et al. in 1987 [6]. The scores were grouped into four categories: 0, 1–2 (mild), 3–4 (moderate), and ≥ 5 (severe); patients with a score of 0 were set as the reference group in the analysis [25]. Statistical analyses were performed using SAS version 9.4 (SAS Institute Inc., Cary, NC, USA) and RStudio version 1.0.136 (R Project for Statistical Computing, Vienna, Austria). *P* values < 0.05 were considered statistically significant.

## Results

### Demographic characteristics of cancer patients in Korea

The demographic characteristics of 2979 cancer patients (50.8% men) diagnosed in 2006 are presented in Table 1. The patients’ mean age was 57.4 (standard deviation, 15.4) years. Among male patients, the most prevalent malignancy was gastric cancer (21.7%), followed by colorectal (15.3%) and liver cancers (12.1%). Among female patients, 31.3% were diagnosed with sex-specific cancers (ICD-10: C50–C63), in which the detailed ICD codes were masked. During the study period, 41.6% of male and 26.6% of female patients had died.

**Table 1:** Demographic characteristics of cancer patients in Korea in 2006, NHIS-NSC

	Total (***n*** = 2979)		Male (***n*** = 1514)		Female (***n*** = 1465)	
	n	%	n	%	n	%
**Age, mean (SD), median**	57.4 (15.4)	59.0	59.1 (15.0)	62.0	55.5 (15.6)	55.0
**Survival time in years, mean (SD), median**	7.1 (3.4)	9.2	6.6 (2.1)	9.0	7.8 (3.1)	9.3
**Sex**						
Male	1514	50.8				
Female	1465	49.2				
**Cause of Death**						
Cancer death	686	23.0	434	28.7	252	17.2
Other cancer death	135	4.5	86	5.7	49	3.3
Non-cancer death	238	8.0	150	9.9	88	6.0
Alive	1920	64.5	844	55.7	1076	73.4
**Cancer type (ICD-10)**						
Stomach (C16)	491	16.5	328	21.7	163	11.01
Colon and rectum (C18-C20)	403	13.5	232	15.3	171	11.7
Liver (C22)	258	8.7	183	12.1	75	5.1
Gallbladder (C23-C24)	60	2.0	25	1.7	35	2.4
Pancreas (C25)	58	1.9	29	1.9	29	2.0
Lung (C33-C34)	232	7.8	157	10.4	75	5.1
Bladder (C67)	71	2.4	61	4.0	10	.7
Thyroid (C73)	295	9.9	54	3.6	241	16.5
Non-Hodgkin lymphoma (C82-C85, C96)	57	1.9	31	2.0	26	1.8
Genital organs ^a)^	592	19.9	133	8.8	459	31.3
Other Cancer ^b)^	462	15.5	281	18.6	181	12.4

*SD* Standard deviation, *NHIS-NSC* National Health Insurance Service-National Sample Cohort

^a)^ Cancer sites are masked and grouped: Breast (C50), Vulva (C51), Vagina (C52), Cervix uteri (C53), Corpus uteri (C54), Uterus unspecified (C55), Ovary (C56), other female genital organs (C57), Placenta (C58) in females and Penis (C60), Prostate (C61), Testis (C62), and other male genital organs (C63) in males

^b)^ “Other cancers” include Lip, oral cavity, and pharynx (C00-C14), Esophagus (C15), Larynx (C32), Kidney (C64), Brain and central nervous system (CNS) (C70-C72), Hodgkin lymphoma (C81), Multiple myeloma (C90), Leukemia (C91-C95), and Other malignant neoplasms (Remainder C00–C97)

### Comorbidity prevalence determined using assessment methods

**Fig. 1:**
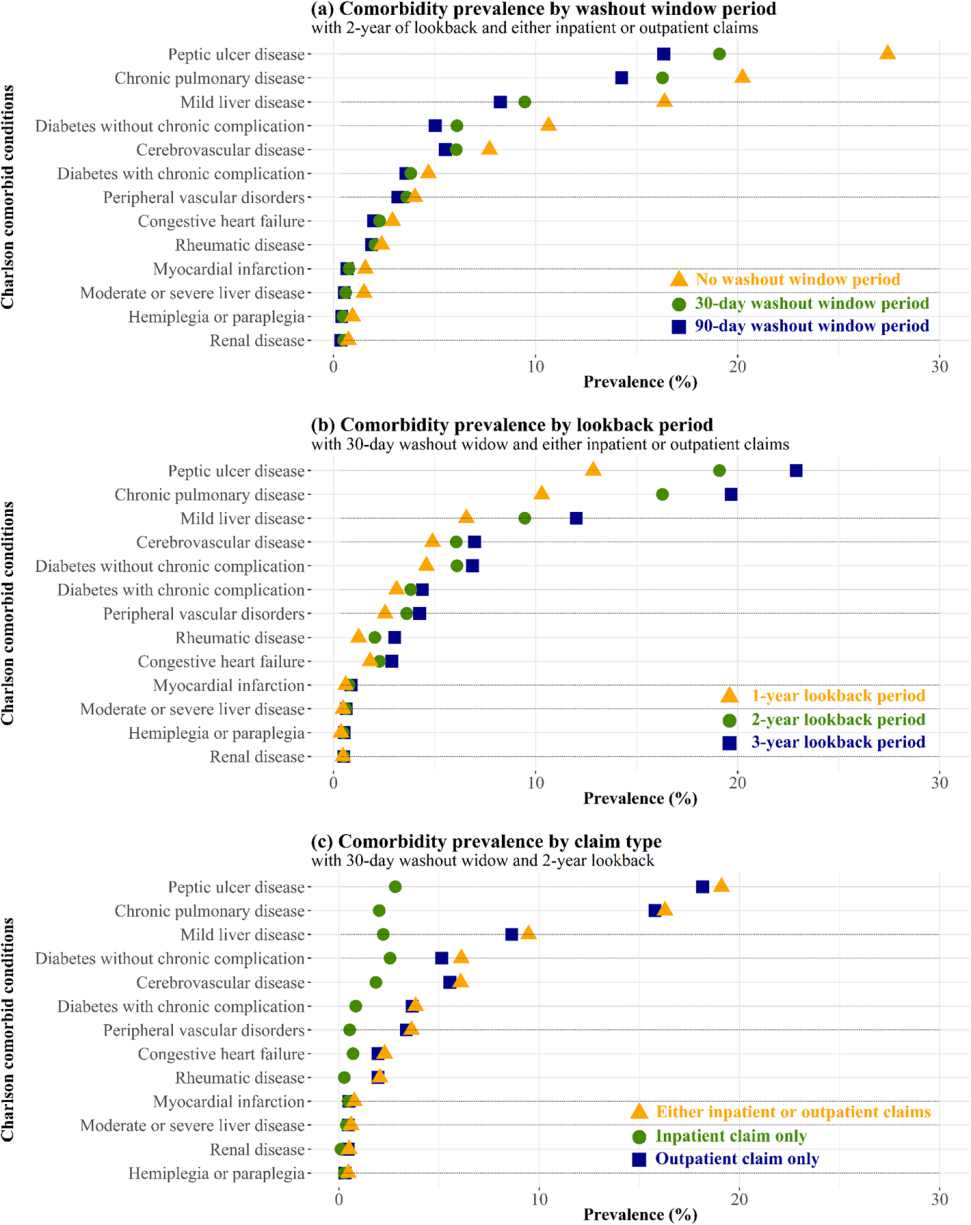
Prevalence of Charlson comorbidities by (**a**) washout window, (**b**) lookback period, and (**c**) claim type

The comorbidity prevalence that resulted from using different methods is presented (Fig. 1, Table S1). The results show peptic ulcer disease (19.1%), chronic pulmonary disease (16.3%), and mild liver disease (9.5%) as the most common conditions affecting cancer patients in general (the prevalence rates were calculated based on either inpatient or outpatient claims, a 30-day washout window, and 2-year lookback period).

The comorbidity prevalence estimates based on a 2-year lookback period and either inpatient or outpatient claims are presented with different washout window periods in Fig. 1a. The prevalence increased considerably when a washout window period was not used (No WP). However, the changes in prevalence from a 90- to 30-day washout window were relatively small compared to a 30-day washout window to No WP. The peptic ulcer prevalence increased by 8.3% if the washout window period was not considered (from 30-day to No WP: 19.1 to 27.4%). Whereas shortening the washout window period from 90-day to 30-day resulted in an increase of only 2.7% (from 90-day to 30-day: 16.4 to 19.1%).

The impact of the lookback period on the comorbidity prevalence estimates, together with a 30-day washout window based on either inpatient or outpatient claims, is demonstrated in Fig. 1b. Peptic ulcers’ prevalence increased by up to 10.0% (from 1-year to 3-year: 12.9 to 22.9%). The difference in prevalence between 1- and 2-year lookback was relatively large compared to the difference between 2- and 3-year lookback for all conditions, except congestive heart failure and rheumatic disease.

The majority of comorbidities were captured from the outpatient claims within a 30-day washout window and 2-year lookback. An analysis of inpatient claims revealed that only 2.8% of patients had peptic ulcer disease; in contrast, an analysis of either inpatient or outpatient claims revealed that 19.1% of patients had this disease (Fig. 1c).

Furthermore, the prevalence of comorbidities observed in inpatient claims increased sharply when No WP was applied but not when a more extended lookback period was used. Specifically, the prevalence of peptic ulcer disease changed from 2.2% (90-day washout window) to 10.2% (No WP) when observed over a 2-year lookback period. However, an increase in lookback from 1 to 3 years resulted in only a 1.4% maximum difference [see Additional file 2].

**Fig. 2:**
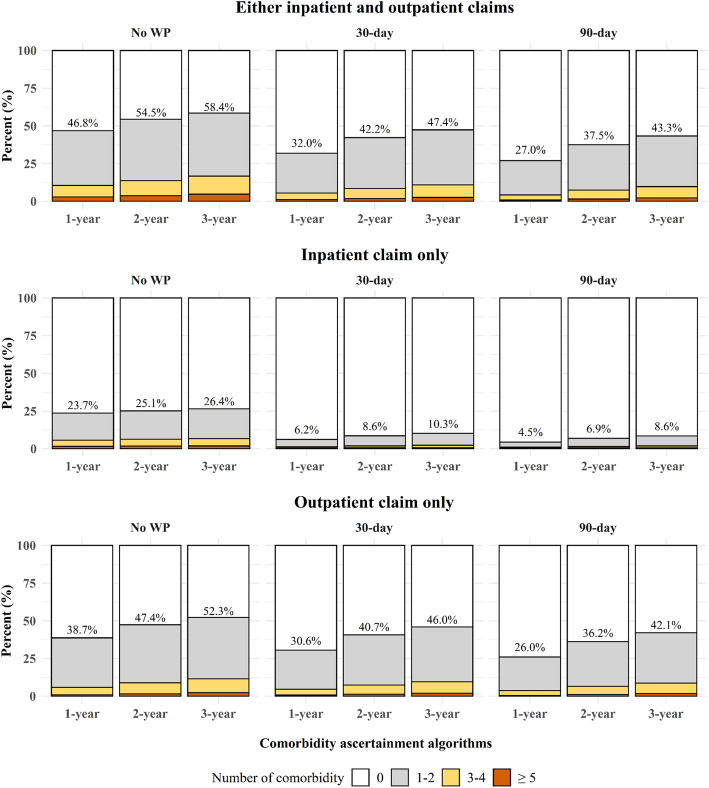
The number of patients with multiple Charlson comorbid conditions by claim types No WP: washout window period was not used

According to different comorbidity assessment methods, changes in the number of comorbid conditions were compared (Fig. 2). With the most prolonged ascertainment period, at least one comorbidity was identified in 58.4% of the patients, whereas, in the analysis using the shortest ascertainment period, comorbidity was identified in only 4.5% of the patients. Although analyses using 30-day and 90-day washout window periods yielded relatively comparable estimates of the total number of conditions, a sharp increase was observed with No WP, mainly when the analysis only included inpatient claims.

**Fig. 3:**
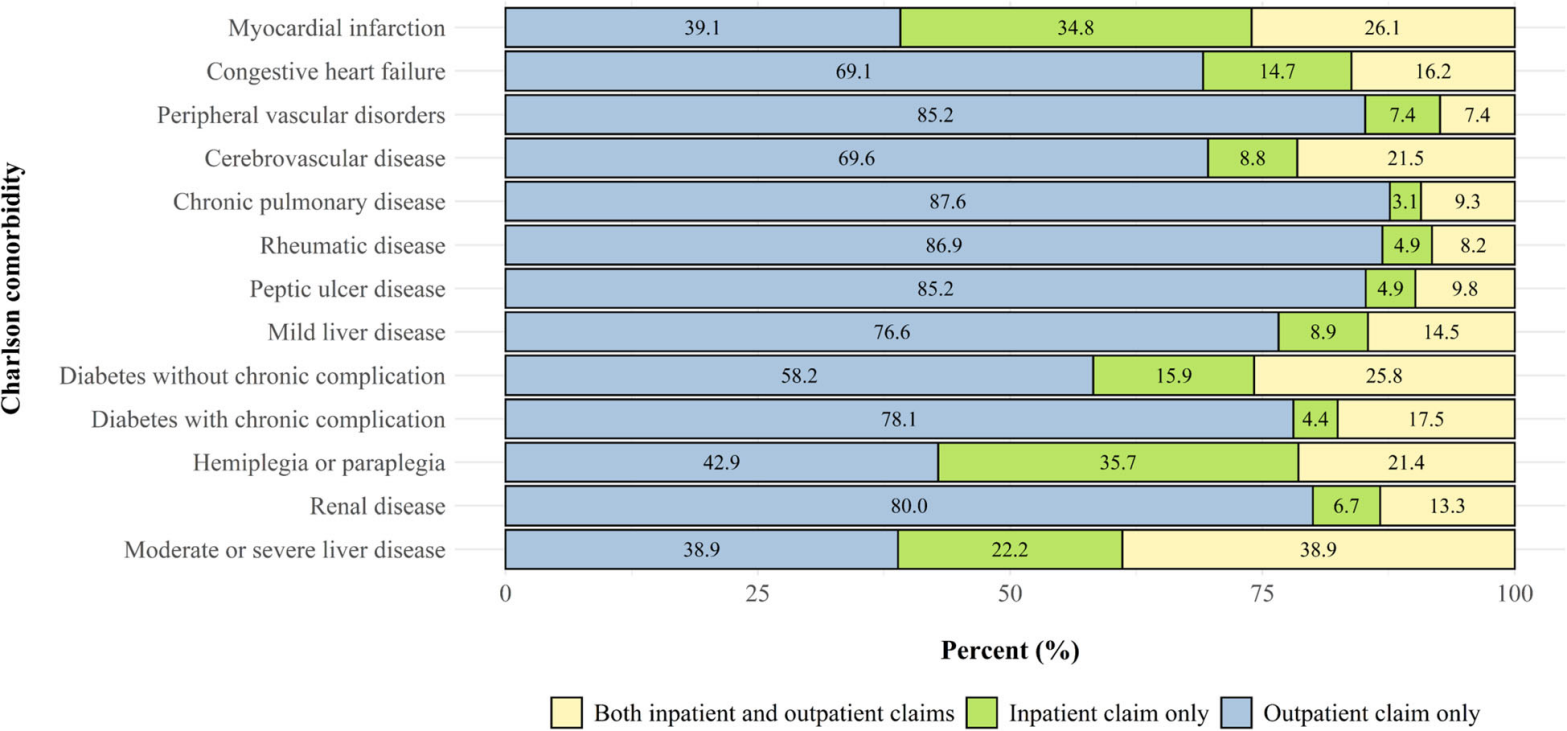
Distribution of claims in each Charlson comorbidity captured with a 30-day washout window and 2-year lookback

Figure 3 illustrates the differences in the distribution of claim types per comorbid condition. When inpatient claims were not used to measure comorbidity, 34.8 and 22.2% of patients with myocardial infarction and moderate or severe liver disease, respectively, were missed. In contrast, less than 5% of patients with chronic pulmonary disease (3.1%), rheumatic disease (4.9%), peptic ulcer disease (4.9%), and diabetes with chronic complications (4.4%) were missed when using outpatient claims only.

### Impact of Charlson comorbidity on non-cancer mortality

The estimated HRs and impacts of each comorbid condition changed according to the use of different combinations of the washout window period, lookback period, and claim types [see Additional file 3]. In the analyses of either inpatient or outpatient claims with a 2-year lookback, the highest risk of non-cancer mortality was associated with moderate or severe liver disease. The HR increased from 5.5 to 9.9 as the washout window period increased. Myocardial infarction captured with No WP showed significant variations in HRs. However, the HRs associated with diabetes without chronic complications showed fewer variations.

The HRs for non-cancer mortality ranged from 1.0 (90-day washout window, 2-year lookback, and outpatient claim only) to 3.0 (90-day washout window, 1-year lookback, and inpatient claims only) among cancer patients with CCI scores of 1–2 (Table 2). Among those with CCI scores of 3–4, the HRs ranged from 1.0 (90-day washout window, 2-year lookback, and outpatient claim only) to 5.0 (30-day washout window, 1-year lookback, and inpatient claims). For those with CCI scores of ≥5, the HRs ranged from 3.7 (No WP, 1-year lookback, and outpatient claims) to 8.0 (No WP, 3-year lookback, and inpatient claims). Using either inpatient or outpatient claims, the HRs decreased gradually as both the washout window and lookback period increased. However, the analysis based on inpatient claims only increased HRs associated with CCI score of 1–2 (mild) and 3–4 (moderate) from 1.7 to 2.2 and 3.8 to 4.9, respectively, with a 30-day washout window and 2-year lookback. In a comparison of the risk estimates based on a 30-day washout window, 2-year lookback, and either inpatient or outpatient claims, the HRs (95% CIs) for non-cancer mortalities were 1.3 (1.0–1.7), 1.7 (1.1–2.6), and 5.5 (3.3–9.1) among cancer patients with CCI scores of 1–2 (mild), 3–4 (moderate), and ≥ 5 (severe), respectively.

**Table 2:** Hazard ratios associated with the Charlson Comorbidity Index according to different assessment methods

Ascertainment period		CCI	***Non-cancer death***					
			Either inpatient or outpatient claims		Inpatient claim only		Outpatient claim only	
Washout window	Lookback		HR	95% CI	HR	95% CI	HR	95% CI
No WP	1-year	1–2	1.9	(1.4, 2.6)	2.0	(1.5, 2.7)	1.5	(1.1, 2.0)
		3–4	3.1	(2.1, 4.6)	3.4	(2.3, 5.1)	2.2	(1.4, 3.4)
		≥5	6.1	(3.9, 9.4)	7.9	(4.9, 12.7)	3.8	(2.0, 7.1)
	2-year	1–2	1.6	(1.2, 2.3)	1.7	(1.3, 2.4)	1.4	(1.0, 1.8)
		3–4	2.6	(1.7, 3.9)	3.8	(2.6, 5.5)	1.6	(1.0, 2.5)
		≥5	5.6	(3.6, 8.6)	7.9	(5.0, 12.5)	4.2	(2.5, 7.1)
	3-year	1–2	1.5	(1.1, 2.2)	1.8	(1.3, 2.4)	1.3	(0.9, 1.7)
		3–4	2.7	(1.8, 4.0)	3.6	(2.5, 5.3)	1.8	(1.2, 2.7)
		≥5	4.9	(3.3, 7.6)	8.0	(5.1, 12.5)	3.7	(2.3, 5.9)
30-day	1-year	1–2	1.6	(1.2, 2.2)	2.7	(1.8, 4.0)	1.5	(1.1, 1.9)
		3–4	2.2	(1.4, 3.5)	5.0	(2.7, 9.3)	1.7	(1.0, 2.9)
		≥5	6.1	(3.5, 10.5)	7.1	(3.6, 14.0)	5.2	(2.7, 10.0)
	2-year	1–2	1.3	(1.0, 1.7)	2.2	(1.5, 3.2)	1.2	(0.9, 1.6)
		3–4	1.7	(1.1, 2.6)	4.9	(2.9, 8.2)	1.2	(0.7, 2.0)
		≥5	5.5	(3.3, 9.1)	7.5	(3.9, 14.3)	5.2	(3.0, 8.9)
	3-year	1–2	1.2	(0.9, 1.6)	2.2	(1.5, 3.2)	1.2	(0.9, 1.5)
		3–4	1.8	(1.2, 2.7)	4.4	(2.7, 7.1)	1.5	(0.9, 2.2)
		≥5	3.6	(2.3, 5.7)	6.7	(3.5, 12.7)	3.7	(2.3, 6.1)
90-day	1-year	1–2	1.5	(1.1, 1.9)	3.0	(1.9, 4.7)	1.3	(0.9, 1.7)
		3–4	1.7	(1.0, 2.8)	4.4	(2.1, 9.0)	1.4	(0.8, 2.4)
		≥5	6.4	(3.5, 11.6)	7.5	(3.8, 14.9)	5.9	(2.6, 13.6)
	2-year	1–2	1.1	(0.8, 1.4)	2.2	(1.5, 3.3)	1.0	(0.7, 1.3)
		3–4	1.3	(0.8, 2.1)	4.5	(2.6, 7.8)	1.0	(0.6, 1.6)
		≥5	5.5	(3.3, 9.1)	7.8	(4.1, 15.1)	4.9	(2.7, 8.7)
	3-year	1–2	1.0	(0.8, 1.4)	2.2	(1.5, 3.2)	1.0	(0.7, 1.3)
		3–4	1.4	(0.9, 2.2)	4.0	(2.4, 6.7)	1.1	(0.7, 1.8)
		≥5	3.8	(2.4, 5.9)	6.9	(3.6, 13.3)	3.7	(2.2, 6.3)

*No WP* Washout window period was not used, *CCI* Charlson Comorbidity Index, *HR* Hazard ratio, *CI* Confidence interval

^a)^ Prognostic prediction models were based on a sex-adjusted Cox proportional hazards model accounting for left truncated and right-censored data

## Discussion

The CCI has been used to measure comorbidity and adjust for associated risks in survival models based on the various data sources, including clinical trials, prospective and retrospective cohort studies, and claims data [26–33]. Recently, population-based health care claims data have been used more frequently in studies of health-related outcomes, as these data could yield generalizable results. However, it remained unclear how comorbid conditions in cancer patients can be measured and accounted for modelling non-cancer mortality based on claims data. This study is the first to systematically compare various comorbidity assessment methods and evaluate their impact on non-cancer mortality risk estimates using cancer patients’ health care claims data. The effects of different washout window periods, lookback periods, and claim types on comorbidity prevalence and associated non-cancer mortality risk were presented.

In the absence of a washout window period, a substantial increase in the prevalence of comorbidities was observed, highlighting the critical role of the washout window period in preventing up-coding claims. Cancer patients may frequently visit the hospital right before a cancer diagnosis, and some diagnostic codes applied to the medical examination may be recorded for administrative purposes. Likewise, complications related to cancer and its treatment should be differentiated from comorbidities, as these do not represent the patient’s general health before a cancer diagnosis.

Regarding the lookback period, 1 year might be insufficient to account for rare comorbid conditions. In contrast, a 3-year lookback might conservatively capture comorbidities. The comorbidities captured in inpatient claims may represent long periods of hospitalization hence associated higher risk of non-cancer mortality. Such differentiation in the analytical approach might better account for disease severity.

This study assessed the impact of comorbidity on the estimates of non-cancer mortality. The cancer-specific mortality risk could not be evaluated because the NHIS-NSC database lacks information about the cancer stage, which has been shown to affect the cancer mortality risk strongly. Analyses that account for the cancer stage to clarify the association between comorbidity and cancer-specific mortality [3] risk remain future studies. Nevertheless, previous studies have shown that the number and severity of comorbidities strongly influence non-cancer mortality risk, with a relatively lesser effect on cancer-specific mortality [2, 4, 34]. The NHIS data confidentially policy masked some comorbid conditions, including dementia and AIDS/HIV. Therefore, the present study could not evaluate the impacts of these conditions on non-cancer mortality risk, which remained a limitation.

## Conclusions

The study findings suggest that the estimates of comorbidity prevalence and its impact on non-cancer mortality risk vary considerably depending on the assessment method used. These discrepancies demonstrate that selecting an optimal approach is critical to an accurate prognostication of cancer patients’ mortality. Researchers should understand the complexity of comorbidity assessments using claims data and select the assessment method with caution.

## Supplementary Information

**Additional file 1.** The assessment methods to identify comorbid conditions in cancer patients using claims-based data.

**Additional file 2.** Prevalence of Charlson comorbidities according to comorbidity assessment methods among Korean cancer patients in 2006.

**Additional file 3.** Estimated hazard ratios for non-cancer death of Charlson comorbidities among Korean cancer patients in 2006.

## Data Availability

This study used de-identified secondary data from the Korean National Health Insurance Service (NHIS) [24], which are available from NHIS upon request and approval (https://nhiss.nhis.or.kr).

## Abbreviations

CCI: Charlson Comorbidity Index
ICD-9-CM: International Classification of Diseases, Ninth Revision, Clinical Modification
NHIS-NSC: National Health Insurance Service-National Sample Cohort
ICD-10: Tenth Revision of the International Statistical Classification of Diseases and Related Health Problems
HR: Hazard ratio
CI: Confidence interval
No WP: Washout window period was not used

## References

[1] Zaorsky NG, Churilla T, Egleston B, Fisher S, Ridge J, Horwitz E, Meyer J (2017). Causes of death among cancer patients. Ann Oncol.

[2] Cho H, Mariotto AB, Mann BS, Klabunde CN, Feuer EJ (2013). Assessing non–cancer-related health status of US cancer patients: other-cause survival and comorbidity prevalence. Am J Epidemiol.

[3] Søgaard M, Thomsen RW, Bossen KS, Sørensen HT, Nørgaard M (2013). The impact of comorbidity on cancer survival: a review. Clin Epidemiol.

[4] Edwards BK, Noone AM, Mariotto AB, Simard EP, Boscoe FP, Henley SJ, Jemal A, Cho H, Anderson RN, Kohler BA (2014). Annual report to the nation on the status of cancer, 1975-2010, featuring prevalence of comorbidity and impact on survival among persons with lung, colorectal, breast, or prostate cancer. Cancer.

[5] Klabunde CN, Harlan LC, Warren JL (2006). Data sources for measuring comorbidity: a comparison of hospital records and medicare claims for cancer patients. Med Care.

[6] Charlson ME, Pompei P, Ales KL, MacKenzie CR (1987). A new method of classifying prognostic comorbidity in longitudinal studies: development and validation. J Chronic Dis.

[7] Deyo RA, Cherkin DC, Ciol MA (1992). Adapting a clinical comorbidity index for use with ICD-9-CM administrative databases. J Clin Epidemiol.

[8] Quan H, Sundararajan V, Halfon P, Fong A, Burnand B, Luthi J-C, Saunders LD, Beck CA, Feasby TE, Ghali WA (2005). Coding algorithms for defining comorbidities in ICD-9-CM and ICD-10 administrative data. Med Care.

[9] Halfon P, Eggli Y, van Melle G, Chevalier J, Wasserfallen J-B, Burnand B (2002). Measuring potentially avoidable hospital readmissions. J Clin Epidemiol.

[10] Sundararajan V, Henderson T, Perry C, Muggivan A, Quan H, Ghali WA (2004). New ICD-10 version of the Charlson comorbidity index predicted in-hospital mortality. J Clin Epidemiol.

[11] Sundararajan V, Quan H, Halfon P, Fushimi K, Luthi J-C, Burnand B, Ghali WA (2007). Information IMCfCH: cross-national comparative performance of three versions of the ICD-10 Charlson index. Med Care.

[12] Klabunde CN, Potosky AL, Legler JM, Warren JL (2000). Development of a comorbidity index using physician claims data. J Clin Epidemiol.

[13] Klabunde CN, Warren JL, Legler JM (2002). Assessing comorbidity using claims data: an overview. Med Care.

[14] Chen JS, Roberts CL, Simpson JM, Ford JB (2011). Use of hospitalisation history (lookback) to determine prevalence of chronic diseases: impact on modelling of risk factors for haemorrhage in pregnancy. BMC Med Res Methodol.

[15] Griffiths RI, O’Malley CD, Herbert RJ, Danese MD (2013). Misclassification of incident conditions using claims data: impact of varying the period used to exclude pre-existing disease. BMC Med Res Methodol.

[16] Czwikla J, Jobski K, Schink T (2017). The impact of the lookback period and definition of confirmatory events on the identification of incident cancer cases in administrative data. BMC Med Res Methodol.

[17] Diop M, Strumpf EC, Datta GD (2018). Measuring colorectal cancer incidence: the performance of an algorithm using administrative health data. BMC Med Res Methodol.

[18] Epping J, Geyer S, Tetzlaff J (2020). The effects of different lookback periods on the sociodemographic structure of the study population and on the estimation of incidence rates: analyses with German claims data. BMC Med Res Methodol.

[19] Zhang JX, Iwashyna TJ, Christakis NA (1999). The performance of different lookback periods and sources of information for Charlson comorbidity adjustment in Medicare claims. Med Care.

[20] Preen DB, CD’Arcy JH, Spilsbury K, Semmens JB, Brameld KJ (2006). Length of comorbidity lookback period affected regression model performance of administrative health data. J Clin Epidemiol.

[21] Klabunde CN, Legler JM, Warren JL, Baldwin L-M, Schrag D (2007). A refined comorbidity measurement algorithm for claims-based studies of breast, prostate, colorectal, and lung cancer patients. Ann Epidemiol.

[22] Kim KH, Ahn LS (2009). A comparative study on comorbidity measurements with lookback period using health insurance database: focused on patients who underwent percutaneous coronary intervention. J Prev Med Public Health.

[23] Kim KH (2016). Comorbidity adjustment in health insurance claim database. Health Policy Manag.

[24] Lee J, Lee JS, Park S-H, Shin SA, Kim K (2017). Cohort profile: the national health insurance service–national sample cohort (NHIS-NSC), South Korea. Int J Epidemiol.

[25] Menendez ME, Neuhaus V, van Dijk CN, Ring D (2014). The Elixhauser comorbidity method outperforms the Charlson index in predicting inpatient death after orthopaedic surgery. Clin Orthop Relat Res.

[26] Park JW, Koh DH, Jang WS, Lee JY, Cho KS, Ham WS, Rha KH, Jung WH, Hong SJ, Choi YD (2018). Age-adjusted Charlson Comorbidity Index as a prognostic factor for radical prostatectomy outcomes of very high-risk prostate cancer patients. PLoS One.

[27] Yoon S-J, Kim E-J, Seo H-J, Oh I-H (2015). The association between Charlson comorbidity index and the medical care cost of cancer: a retrospective study. Biomed Res Int.

[28] Woo HK, Park JH, Kang HS, Kim SY, Lee SI, Nam HH (2010). Charlson comorbidity index as a predictor of long-term survival after surgery for breast cancer: a nationwide retrospective cohort study in South Korea. J Breast Cancer.

[29] Kang S, Kim H-S, Kim W, Kim JH, Kang SH, Han I (2015). Comorbidity is independently associated with poor outcome in extremity soft tissue sarcoma. Clin Orthoped Surg.

[30] Lee SY, Kang EJ, Lee SY, Kim HJ, Min KH, Hur GY, Shim JJ, Kang KH, Oh SC, Seo JH (2018). Efficacy of second-line treatment and importance of comorbidity scores and clinical parameters affecting prognosis in elderly patients with non-small cell lung cancer without epidermal growth factor receptor mutations. Oncol Lett.

[31] Park BR, Kim SY, Shin DW, Yang HK, Park JH (2017). Influence of socioeconomic status, comorbidity, and disability on late-stage cancer diagnosis. Osong Public Health Res Perspect.

[32] Bang JH, Hwang S-H, Lee E-J, Kim Y (2013). The predictability of claim-data-based comorbidity-adjusted models could be improved by using medication data. BMC Med Inform Decis Mak.

[33] Hwang SM, Yoon SJ, Ahn HS, An HG, Kim SH, Kyeong MH, Lee EK (2009). Usefulness of comorbidity indices in operative gastric cancer cases. J Prev Med Public Health.

[34] Jørgensen T, Hallas J, Friis S, Herrstedt J (2012). Comorbidity in elderly cancer patients in relation to overall and cancer-specific mortality. Br J Cancer.

