# Supplementary material for: Impact of comorbidity assessment methods to predict non-cancer mortality risk in cancer patients: a retrospective observational study using the National Health Insurance Service claims-based data in Korea
Source: BMC Med Res Methodol. 2021 Apr 9;21:66. doi: 10.1186/s12874-021-01257-2 (PMC8035736; doi:10.1186/s12874-021-01257-2)
Supplement: Supplementary file 1 — Additional file 1. The assessment methods to identify comorbid conditions in cancer patients using claims-based data. [file 12874_2021_1257_MOESM1_ESM.docx]

Additional file 1. The assessment methods to identify comorbid conditions in patients with cancer using claims-based data


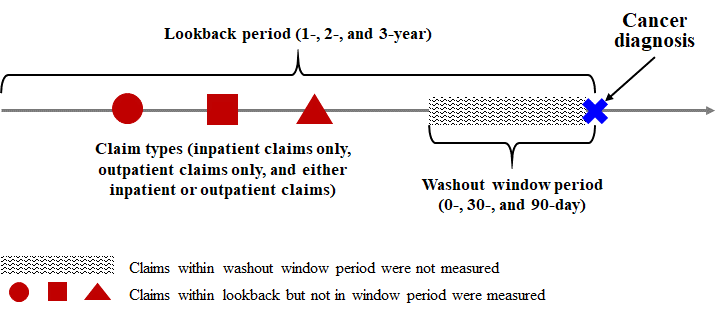


|  |  |  |  |  |  | **Ascertainment period** | | | |
| --- | --- | --- | --- | --- | --- | --- | --- | --- | --- |
|  |  |  |  |  |  |  | | | |
|  |  |  |  |  |  | **Washout window period** |  |  | **Lookback period** |
|  |  |  | **Claim types** |  |  |  |  |  |  |
|  |  |  |  |  |  |  |  |  |  |
|  |  |  |  |  |  |  |  |  |  |
|  |  |  |  |  |  | **No WP** |  |  | **1-year** |
|  |  |  |  |  |  |  |  |  |  |
|  |  |  |  |  |  |  |  |  | **2-year** |
|  |  |  |  |  |  |  |  |  |  |
|  |  |  |  |  |  |  |  |  | **3-year** |
|  |  |  |  |  |  |  |  |  |  |
|  |  |  | **Either inpatient or outpatient claims** |  |  | **30-day** |  |  | **1-year** |
|  |  |  |  |  |  |  |  |  |  |
|  |  |  |  |  |  |  |  |  | **2-year** |
|  |  |  |  |  |  |  |  |  |  |
|  |  |  |  |  |  |  |  |  | **3-year** |
|  |  |  |  |  |  |  |  |  |  |
|  |  |  |  |  |  | **90-day** |  |  | **1-year** |
|  |  |  |  |  |  |  |  |  |  |
|  |  |  |  |  |  |  |  |  | **2-year** |
|  |  |  |  |  |  |  |  |  |  |
|  |  |  |  |  |  |  |  |  | **3-year** |
|  |  |  |  |  |  |  |  |  |  |
| **2,979 Korean cancer patients in 2006** |  |  |  |  |  | **No WP** |  |  | **1-year** |
|  |  |  |  |  |  |  |  |  |  |
|  |  |  |  |  |  |  |  |  | **2-year** |
|  |  |  |  |  |  |  |  |  |  |
|  |  |  |  |  |  |  |  |  | **3-year** |
|  |  |  |  |  |  |  |  |  |  |
|  |  |  | **Inpatient**  **claims only** |  |  | **30-day** |  |  | **1-year** |
|  |  |  |  |  |  |  |  |  |  |
|  |  |  |  |  |  |  |  |  | **2-year** |
|  |  |  |  |  |  |  |  |  |  |
|  |  |  |  |  |  |  |  |  | **3-year** |
|  |  |  |  |  |  |  |  |  |  |
|  |  |  |  |  |  | **90-day** |  |  | **1-year** |
|  |  |  |  |  |  |  |  |  |  |
|  |  |  |  |  |  |  |  |  | **2-year** |
|  |  |  |  |  |  |  |  |  |  |
|  |  |  |  |  |  |  |  |  | **3-year** |
|  |  |  |  |  |  |  |  |  |  |
|  |  |  |  |  |  | **No WP** |  |  | **1-year** |
|  |  |  |  |  |  |  |  |  |  |
|  |  |  |  |  |  |  |  |  | **2-year** |
|  |  |  |  |  |  |  |  |  |  |
|  |  |  |  |  |  |  |  |  | **3-year** |
|  |  |  |  |  |  |  |  |  |  |
|  |  |  | **Outpatient**  **claims only** |  |  | **30-day** |  |  | **1-year** |
|  |  |  |  |  |  |  |  |  |  |
|  |  |  |  |  |  |  |  |  | **2-year** |
|  |  |  |  |  |  |  |  |  |  |
|  |  |  |  |  |  |  |  |  | **3-year** |
|  |  |  |  |  |  |  |  |  |  |
|  |  |  |  |  |  | **90-day** |  |  | **1-year** |
|  |  |  |  |  |  |  |  |  |  |
|  |  |  |  |  |  |  |  |  | **2-year** |
|  |  |  |  |  |  |  |  |  |  |
|  |  |  |  |  |  |  |  |  | **3-year** |
