# Supplementary material for: Impact of comorbidity assessment methods to predict non-cancer mortality risk in cancer patients: a retrospective observational study using the National Health Insurance Service claims-based data in Korea
Source: BMC Med Res Methodol. 2021 Apr 9;21:66. doi: 10.1186/s12874-021-01257-2 (PMC8035736; doi:10.1186/s12874-021-01257-2)
Supplement: Supplementary file 2 — Additional file 2. Prevalence of Charlson comorbidities according to comorbidity assessment methods among Korean cancer patients in 2006. [file 12874_2021_1257_MOESM2_ESM.docx]

Additional file 2. Prevalence of Charlson comorbidities according to comorbidity assessment methods among Korean cancer patients in 2006

|  |  | **No washout window** | | | | | | **30-day washout window** | | | | | | **90-day washout window** | | | | | |
| --- | --- | --- | --- | --- | --- | --- | --- | --- | --- | --- | --- | --- | --- | --- | --- | --- | --- | --- | --- |
|  |  | **1-year lookback** | | **2-year lookback** | | **3-year lookback** | | **1-year lookback** | | **2-year lookback** | | **3-year lookback** | | **1-year lookback** | | **2-year lookback** | | **3-year lookback** | |
|  | **Conditions in the CCI** | **n** | **%** | **n** | **%** | **n** | **%** | **n** | **%** | **n** | **%** | **n** | **%** | **n** | **%** | **n** | **%** | **n** | **%** |
| Either inpatient and outpatient claims | Myocardial infarction | 42 | 1.41 | 47 | 1.58 | 50 | 1.68 | 18 | .60 | 23 | .77 | 26 | .87 | 14 | .47 | 20 | .67 | 23 | .77 |
|  | Congestive heart failure | 74 | 2.48 | 87 | 2.92 | 106 | 3.56 | 54 | 1.81 | 68 | 2.28 | 86 | 2.89 | 44 | 1.48 | 59 | 1.98 | 76 | 2.55 |
|  | Peripheral vascular disorders | 92 | 3.09 | 120 | 4.03 | 140 | 4.70 | 76 | 2.55 | 108 | 3.63 | 127 | 4.26 | 61 | 2.05 | 95 | 3.19 | 115 | 3.86 |
|  | Cerebrovascular disease | 199 | 6.68 | 230 | 7.72 | 252 | 8.46 | 146 | 4.90 | 181 | 6.08 | 208 | 6.98 | 126 | 4.23 | 165 | 5.54 | 191 | 6.41 |
|  | Chronic pulmonary disease | 439 | 14.74 | 603 | 20.24 | 693 | 23.26 | 307 | 10.31 | 485 | 16.28 | 586 | 19.67 | 250 | 8.39 | 425 | 14.27 | 537 | 18.03 |
|  | Rheumatic disease | 47 | 1.58 | 71 | 2.38 | 98 | 3.29 | 37 | 1.24 | 61 | 2.05 | 90 | 3.02 | 33 | 1.11 | 56 | 1.88 | 84 | 2.82 |
|  | Peptic ulcer disease | 656 | 22.02 | 817 | 27.43 | 923 | 30.98 | 383 | 12.86 | 569 | 19.10 | 682 | 22.89 | 285 | 9.57 | 487 | 16.35 | 606 | 20.34 |
|  | Mild liver disease | 396 | 13.29 | 488 | 16.38 | 561 | 18.83 | 196 | 6.58 | 282 | 9.47 | 358 | 12.02 | 164 | 5.51 | 246 | 8.26 | 319 | 10.71 |
|  | Diabetes without chronic complication | 283 | 9.50 | 317 | 10.64 | 335 | 11.25 | 137 | 4.60 | 182 | 6.11 | 205 | 6.88 | 107 | 3.59 | 150 | 5.04 | 177 | 5.94 |
|  | Diabetes with chronic complication | 119 | 3.99 | 140 | 4.70 | 155 | 5.20 | 93 | 3.12 | 114 | 3.83 | 131 | 4.40 | 85 | 2.85 | 107 | 3.59 | 123 | 4.13 |
|  | Hemiplegia or paraplegia | 25 | .84 | 28 | .94 | 29 | .97 | 11 | .37 | 14 | .47 | 16 | .54 | 9 | .30 | 12 | .40 | 14 | .47 |
|  | Renal disease | 21 | .70 | 22 | .74 | 22 | .74 | 14 | .47 | 15 | .50 | 15 | .50 | 10 | .34 | 11 | .37 | 11 | .37 |
|  | Moderate or severe liver disease | 41 | 1.38 | 45 | 1.51 | 46 | 1.54 | 14 | .47 | 18 | .60 | 19 | .64 | 11 | .37 | 16 | .54 | 17 | .57 |
| Inpatient claim only | Myocardial infarction | 34 | 1.14 | 40 | 1.34 | 41 | 1.38 | 8 | .27 | 14 | .47 | 15 | .50 | 5 | .17 | 11 | .37 | 12 | .40 |
|  | Congestive heart failure | 40 | 1.34 | 45 | 1.51 | 48 | 1.61 | 15 | .50 | 21 | .70 | 24 | .81 | 9 | .30 | 15 | .50 | 18 | .60 |
|  | Peripheral vascular disorders | 21 | .70 | 27 | .91 | 28 | .94 | 8 | .27 | 16 | .54 | 17 | .57 | 5 | .17 | 13 | .44 | 14 | .47 |
|  | Cerebrovascular disease | 103 | 3.46 | 114 | 3.83 | 127 | 4.26 | 37 | 1.24 | 55 | 1.85 | 70 | 2.35 | 27 | .91 | 45 | 1.51 | 60 | 2.01 |
|  | Chronic pulmonary disease | 181 | 6.08 | 193 | 6.48 | 203 | 6.81 | 47 | 1.58 | 60 | 2.01 | 71 | 2.38 | 35 | 1.17 | 48 | 1.61 | 59 | 1.98 |
|  | Rheumatic disease | 11 | .37 | 15 | .50 | 17 | .57 | - | - | 8 | .27 | 10 | .34 | - | - | 7 | .23 | 9 | .30 |
|  | Peptic ulcer disease | 287 | 9.63 | 303 | 10.17 | 320 | 10.74 | 64 | 2.15 | 84 | 2.82 | 105 | 3.52 | 44 | 1.48 | 64 | 2.15 | 85 | 2.85 |
|  | Mild liver disease | 240 | 8.06 | 252 | 8.46 | 265 | 8.90 | 50 | 1.68 | 66 | 2.22 | 80 | 2.69 | 35 | 1.17 | 51 | 1.71 | 65 | 2.18 |
|  | Diabetes without chronic complication | 211 | 7.08 | 225 | 7.55 | 235 | 7.89 | 56 | 1.88 | 76 | 2.55 | 87 | 2.92 | 37 | 1.24 | 57 | 1.91 | 68 | 2.28 |
|  | Diabetes with chronic complication | 49 | 1.64 | 56 | 1.88 | 60 | 2.01 | 17 | .57 | 25 | .84 | 29 | .97 | 14 | .47 | 22 | .74 | 26 | .87 |
|  | Hemiplegia or paraplegia | 19 | .64 | 21 | .70 | 21 | .70 | 6 | .20 | 8 | .27 | 9 | .30 | 5 | .17 | 7 | .23 | 8 | .27 |
|  | Renal disease | 11 | .37 | 11 | .37 | 11 | .37 | - | - | - | - | - | - | - | - | - | - | - | - |
|  | Moderate or severe liver disease | 34 | 1.14 | 36 | 1.21 | 36 | 1.21 | 9 | .30 | 11 | .37 | 11 | .37 | 7 | .23 | 9 | .30 | 9 | .30 |
| Outpatient claim only | Myocardial infarction | 15 | .50 | 16 | .54 | 18 | .60 | 14 | .47 | 15 | .50 | 17 | .57 | 12 | .40 | 14 | .47 | 16 | .54 |
|  | Congestive heart failure | 51 | 1.71 | 63 | 2.11 | 81 | 2.72 | 46 | 1.54 | 58 | 1.95 | 75 | 2.52 | 40 | 1.34 | 53 | 1.78 | 69 | 2.32 |
|  | Peripheral vascular disorders | 76 | 2.55 | 103 | 3.46 | 123 | 4.13 | 70 | 2.35 | 100 | 3.36 | 118 | 3.96 | 56 | 1.88 | 87 | 2.92 | 106 | 3.56 |
|  | Cerebrovascular disease | 152 | 5.10 | 182 | 6.11 | 204 | 6.85 | 135 | 4.53 | 165 | 5.54 | 190 | 6.38 | 117 | 3.93 | 151 | 5.07 | 175 | 5.87 |
|  | Chronic pulmonary disease | 355 | 11.92 | 527 | 17.69 | 626 | 21.01 | 294 | 9.87 | 470 | 15.78 | 573 | 19.23 | 241 | 8.09 | 410 | 13.76 | 524 | 17.59 |
|  | Rheumatic disease | 40 | 1.34 | 63 | 2.11 | 91 | 3.05 | 35 | 1.17 | 58 | 1.95 | 86 | 2.89 | 32 | 1.07 | 54 | 1.81 | 81 | 2.72 |
|  | Peptic ulcer disease | 517 | 17.35 | 684 | 22.96 | 798 | 26.79 | 356 | 11.95 | 541 | 18.16 | 650 | 21.82 | 267 | 8.96 | 466 | 15.64 | 580 | 19.47 |
|  | Mild liver disease | 249 | 8.36 | 345 | 11.58 | 419 | 14.07 | 173 | 5.81 | 257 | 8.63 | 326 | 10.94 | 148 | 4.97 | 226 | 7.59 | 292 | 9.80 |
|  | Diabetes without chronic complication | 141 | 4.73 | 179 | 6.01 | 202 | 6.78 | 116 | 3.89 | 153 | 5.14 | 176 | 5.91 | 94 | 3.16 | 130 | 4.36 | 155 | 5.20 |
|  | Diabetes with chronic complication | 97 | 3.26 | 118 | 3.96 | 133 | 4.46 | 91 | 3.05 | 109 | 3.66 | 125 | 4.20 | 83 | 2.79 | 102 | 3.42 | 117 | 3.93 |
|  | Hemiplegia or paraplegia | 10 | .34 | 12 | .40 | 13 | .44 | 7 | .23 | 9 | .30 | 10 | .34 | 6 | .20 | 8 | .27 | 9 | .30 |
|  | Renal disease | 15 | .50 | 16 | .54 | 16 | .54 | 13 | .44 | 14 | .47 | 14 | .47 | 9 | .30 | 10 | .34 | 10 | .34 |
|  | Moderate or severe liver disease | 14 | .47 | 17 | .57 | 18 | .60 | 11 | .37 | 14 | .47 | 15 | .50 | 9 | .30 | 13 | .44 | 14 | .47 |

CCI, Charlson Comorbidity Index

Cases < 5 were marked as ‘-’ sign.
