# Supplementary material for: Impact of comorbidity assessment methods to predict non-cancer mortality risk in cancer patients: a retrospective observational study using the National Health Insurance Service claims-based data in Korea
Source: BMC Med Res Methodol. 2021 Apr 9;21:66. doi: 10.1186/s12874-021-01257-2 (PMC8035736; doi:10.1186/s12874-021-01257-2)
Supplement: Supplementary file 3 — Additional file 3. Estimated hazard ratios for non-cancer death of Charlson comorbidities among Korean cancer patients in 2006. [file 12874_2021_1257_MOESM3_ESM.docx]

Additional file 3. Estimated hazard ratios for non-cancer death of Charlson comorbidities among Korean cancer patients in 2006 ^a)^

|  |  | **No washout window** | | | | | | **30-day washout window** | | | | | | **90-day washout window** | | | | | |
| --- | --- | --- | --- | --- | --- | --- | --- | --- | --- | --- | --- | --- | --- | --- | --- | --- | --- | --- | --- |
| **Claim type** |  | **1-year lookback** | | **2-year lookback** | | **3-year lookback** | | **1-year lookback** | | **2-year lookback** | | **3-year lookback** | | **1-year lookback** | | **2-year lookback** | | **3-year lookback** | |
|  | **CCI** | **HR** | **95% CI** | **HR** | **95% CI** | **HR** | **95% CI** | **HR** | **95% CI** | **HR** | **95% CI** | **HR** | **95% CI** | **HR** | **95% CI** | **HR** | **95% CI** | **HR** | **95% CI** |
| Either inpatient or outpatient claims | MI | 1.95 | (1.00, 3.78) | 1.87 | (0.98, 3.59) | 1.59 | (0.84, 3.03) | 1.34 | (0.54, 3.34) | 1.46 | (0.65, 3.32) | 1.17 | (0.52, 2.63) | 1.63 | (0.62, 4.32) | 1.70 | (0.73, 4.00) | 1.25 | (0.54, 2.90) |
|  | CHF | 1.17 | (0.68, 2.01) | 1.11 | (0.67, 1.86) | 1.27 | (0.80, 2.01) | 1.16 | (0.62, 2.16) | 1.00 | (0.56, 1.80) | 1.17 | (0.69, 1.99) | 1.20 | (0.59, 2.45) | 0.97 | (0.51, 1.84) | 1.12 | (0.63, 1.99) |
|  | PVD | 0.71 | (0.38, 1.30) | 0.75 | (0.43, 1.32) | 0.91 | (0.55, 1.50) | 0.77 | (0.39, 1.53) | 0.84 | (0.46, 1.54) | 1.02 | (0.60, 1.73) | 0.75 | (0.34, 1.63) | 0.92 | (0.49, 1.73) | 1.09 | (0.63, 1.88) |
|  | CVD | 1.33 | (0.91, 1.94) | 1.12 | (0.76, 1.65) | 1.11 | (0.77, 1.61) | 1.11 | (0.72, 1.71) | 1.06 | (0.70, 1.61) | 1.07 | (0.72, 1.60) | 1.03 | (0.63, 1.66) | 0.96 | (0.61, 1.52) | 0.98 | (0.63, 1.51) |
|  | COPD | 1.26 | (0.92, 1.74) | 1.16 | (0.86, 1.55) | 1.02 | (0.76, 1.37) | 1.14 | (0.80, 1.63) | 1.02 | (0.74, 1.40) | 0.94 | (0.68, 1.28) | 1.01 | (0.69, 1.50) | 0.94 | (0.67, 1.33) | 0.91 | (0.65, 1.27) |
|  | Rheumatic disease | 1.01 | (0.41, 2.46) | 1.19 | (0.61, 2.34) | 0.92 | (0.49, 1.75) | 1.23 | (0.50, 3.01) | 1.44 | (0.72, 2.87) | 1.03 | (0.53, 1.99) | 1.28 | (0.52, 3.15) | 1.61 | (0.81, 3.21) | 1.18 | (0.61, 2.27) |
|  | PUD | 1.16 | (0.87, 1.55) | 1.08 | (0.82, 1.42) | 1.10 | (0.84, 1.45) | 1.19 | (0.85, 1.69) | 1.00 | (0.74, 1.37) | 0.94 | (0.69, 1.27) | 1.15 | (0.79, 1.68) | 0.91 | (0.65, 1.26) | 0.91 | (0.67, 1.25) |
|  | Mild LD | 2.07 | (1.51, 2.84) | 1.74 | (1.28, 2.36) | 1.89 | (1.41, 2.54) | 2.40 | (1.58, 3.64) | 1.81 | (1.24, 2.65) | 1.98 | (1.40, 2.82) | 2.19 | (1.38, 3.48) | 1.69 | (1.12, 2.53) | 1.81 | (1.25, 2.62) |
|  | Diabetes without C.Cx | 1.19 | (0.83, 1.70) | 1.25 | (0.89, 1.77) | 1.20 | (0.85, 1.70) | 1.59 | (1.04, 2.43) | 1.40 | (0.95, 2.06) | 1.50 | (1.02, 2.19) | 1.21 | (0.72, 2.03) | 1.07 | (0.68, 1.68) | 1.19 | (0.77, 1.83) |
|  | Diabetes with C.Cx | 1.75 | (1.11, 2.76) | 1.89 | (1.22, 2.92) | 1.92 | (1.27, 2.92) | 1.48 | (0.86, 2.54) | 1.67 | (1.03, 2.70) | 1.55 | (0.98, 2.47) | 1.47 | (0.82, 2.63) | 1.84 | (1.14, 2.98) | 1.75 | (1.09, 2.81) |
|  | Hemiplegia, paraplegia | 2.52 | (1.13, 5.63) | 3.12 | (1.48, 6.56) | 3.64 | (1.79, 7.40) | 2.74 | (0.94, 8.00) | 3.70 | (1.48, 9.26) | 3.80 | (1.63, 8.84) | 2.21 | (0.64, 7.60) | 3.15 | (1.14, 8.67) | 3.30 | (1.32, 8.26) |
|  | Renal disease | 2.13 | (0.97, 4.67) | 2.12 | (0.97, 4.63) | 2.08 | (0.96, 4.55) | 1.56 | (0.55, 4.43) | 1.61 | (0.57, 4.53) | 1.62 | (0.58, 4.53) | 1.51 | (0.35, 6.44) | 2.35 | (0.70, 7.85) | 2.29 | (0.69, 7.57) |
|  | Moderate or severe LD | 4.73 | (2.75, 8.16) | 5.50 | (3.32, 9.10) | 4.93 | (2.99, 8.11) | 4.89 | (2.22, 11.27) | 6.95 | (3.45, 13.98) | 6.19 | (3.09, 12.4) | 7.01 | (2.82, 17.7) | 9.85 | (4.92, 19.73) | 7.56 | (3.75, 15.27) |
| Inpatient claim only | MI | 2.41 | (1.13, 5.12) | 2.32 | (1.18, 4.59) | 2.42 | (1.23, 4.76) | 1.41 | (0.30, 6.62) | 1.66 | (0.59, 4.71) | 1.88 | (0.67, 5.28) | 4.53 | (0.73, 28.08) | 2.37 | (0.82, 6.86) | 2.65 | (0.92, 7.54) |
|  | CHF | 1.19 | (0.59, 2.38) | 1.28 | (0.67, 2.48) | 1.34 | (0.72, 2.53) | 0.97 | (0.25, 3.78) | 1.05 | (0.38, 2.89) | 1.10 | (0.42, 2.85) | 0.58 | (0.07, 4.73) | 1.06 | (0.31, 3.67) | 1.20 | (0.38, 3.79) |
|  | PVD | 0.87 | (0.35, 2.16) | 0.94 | (0.40, 2.22) | 0.97 | (0.42, 2.25) | 1.41 | (0.29, 6.87) | 1.98 | (0.62, 6.35) | 1.94 | (0.61, 6.17) | 2.73 | (0.32, 23.48) | 2.62 | (0.67, 10.23) | 2.44 | (0.65, 9.19) |
|  | CVD | 1.66 | (1.03, 2.64) | 1.45 | (0.93, 2.28) | 1.44 | (0.93, 2.23) | 1.42 | (0.68, 2.97) | 1.64 | (0.92, 2.92) | 1.52 | (0.87, 2.66) | 0.97 | (0.36, 2.64) | 1.34 | (0.68, 2.64) | 1.24 | (0.65, 2.36) |
|  | COPD | 1.49 | (0.99, 2.25) | 1.45 | (0.97, 2.17) | 1.42 | (0.95, 2.11) | 1.47 | (0.72, 2.96) | 1.36 | (0.71, 2.62) | 1.34 | (0.72, 2.47) | 1.49 | (0.63, 3.52) | 1.23 | (0.56, 2.72) | 1.21 | (0.59, 2.51) |
|  | Rheumatic disease | 1.13 | (0.14, 8.89) | 1.03 | (0.13, 7.87) | 1.76 | (0.41, 7.47) | - | - | - | - | - | - | - | - | - | - | - | - |
|  | PUD | 1.08 | (0.74, 1.58) | 1.06 | (0.73, 1.53) | 1.01 | (0.70, 1.45) | 1.81 | (1.03, 3.18) | 1.35 | (0.78, 2.33) | 1.29 | (0.77, 2.15) | 1.90 | (0.98, 3.69) | 1.24 | (0.66, 2.33) | 1.18 | (0.67, 2.10) |
|  | Mild LD | 1.89 | (1.29, 2.75) | 1.78 | (1.22, 2.58) | 1.95 | (1.36, 2.80) | 3.81 | (1.74, 8.38) | 3.65 | (1.91, 6.95) | 3.87 | (2.17, 6.90) | 3.50 | (1.39, 8.79) | 3.51 | (1.75, 7.04) | 3.83 | (2.07, 7.09) |
|  | Diabetes without C.Cx | 1.24 | (0.84, 1.83) | 1.38 | (0.95, 1.99) | 1.36 | (0.95, 1.95) | 1.74 | (1.01, 3.00) | 1.78 | (1.11, 2.85) | 1.63 | (1.01, 2.62) | 1.46 | (0.75, 2.84) | 1.61 | (0.92, 2.83) | 1.41 | (0.80, 2.47) |
|  | Diabetes with C.Cx | 2.25 | (1.26, 4.02) | 2.13 | (1.20, 3.77) | 1.92 | (1.09, 3.39) | 1.59 | (0.56, 4.51) | 1.13 | (0.41, 3.09) | 0.76 | (0.27, 2.18) | 1.64 | (0.51, 5.25) | 1.06 | (0.36, 3.15) | 0.74 | (0.24, 2.33) |
|  | Hemiplegia, paraplegia | 1.29 | (0.49, 3.40) | 1.54 | (0.61, 3.85) | 1.49 | (0.59, 3.74) | 0.98 | (0.19, 5.12) | 1.10 | (0.28, 4.29) | 1.09 | (0.29, 4.10) | 0.88 | (0.14, 5.75) | 1.20 | (0.28, 5.14) | 1.25 | (0.31, 5.04) |
|  | Renal disease | 2.43 | (0.94, 6.24) | 2.46 | (0.96, 6.29) | 2.47 | (0.97, 6.32) | 1.57 | (0.19, 13.0) | 1.39 | (0.17, 11.05) | 1.51 | (0.19, 11.9) | 2.32 | (0.26, 20.77) | 1.58 | (0.19, 12.88) | 1.69 | (0.21, 13.63) |
|  | Moderate or severe LD | 4.86 | (2.61, 9.02) | 5.17 | (2.88, 9.26) | 5.12 | (2.87, 9.13) | 2.73 | (0.81, 9.14) | 3.83 | (1.45, 10.10) | 4.01 | (1.58, 10.17) | 5.02 | (1.34, 18.77) | 6.23 | (2.32, 16.71) | 6.63 | (2.59, 17.01) |
| Outpatient claim only | MI | 1.38 | (0.49, 3.89) | 1.42 | (0.51, 3.93) | 1.10 | (0.42, 2.92) | 1.59 | (0.57, 4.44) | 1.62 | (0.59, 4.41) | 1.32 | (0.50, 3.45) | 1.58 | (0.54, 4.63) | 1.53 | (0.54, 4.43) | 1.19 | (0.44, 3.20) |
|  | CHF | 0.76 | (0.37, 1.56) | 0.69 | (0.35, 1.35) | 0.85 | (0.47, 1.53) | 0.83 | (0.40, 1.72) | 0.73 | (0.37, 1.46) | 0.84 | (0.45, 1.57) | 0.97 | (0.44, 2.21) | 0.79 | (0.38, 1.64) | 0.88 | (0.45, 1.71) |
|  | PVD | 0.61 | (0.28, 1.33) | 0.62 | (0.31, 1.23) | 0.87 | (0.50, 1.53) | 0.58 | (0.25, 1.33) | 0.60 | (0.29, 1.24) | 0.82 | (0.45, 1.48) | 0.40 | (0.13, 1.28) | 0.57 | (0.25, 1.30) | 0.81 | (0.42, 1.55) |
|  | CVD | 1.07 | (0.70, 1.64) | 1.03 | (0.68, 1.58) | 1.03 | (0.69, 1.53) | 1.01 | (0.65, 1.59) | 1.05 | (0.68, 1.62) | 1.02 | (0.68, 1.55) | 0.98 | (0.59, 1.62) | 1.01 | (0.63, 1.62) | 1.00 | (0.65, 1.56) |
|  | COPD | 1.11 | (0.79, 1.57) | 1.06 | (0.78, 1.44) | 1.02 | (0.75, 1.38) | 1.13 | (0.79, 1.63) | 1.03 | (0.75, 1.42) | 0.99 | (0.73, 1.36) | 0.99 | (0.66, 1.48) | 0.89 | (0.63, 1.27) | 0.92 | (0.66, 1.28) |
|  | Rheumatic disease | 1.28 | (0.51, 3.17) | 1.52 | (0.76, 3.02) | 1.08 | (0.57, 2.08) | 1.22 | (0.50, 3.00) | 1.56 | (0.79, 3.08) | 1.19 | (0.62, 2.29) | - | - | 1.66 | (0.84, 3.30) | 1.32 | (0.69, 2.53) |
|  | PUD | 1.22 | (0.90, 1.65) | 1.11 | (0.84, 1.48) | 1.14 | (0.86, 1.50) | 1.16 | (0.81, 1.65) | 1.03 | (0.75, 1.41) | 0.95 | (0.70, 1.30) | 1.10 | (0.74, 1.64) | 0.91 | (0.65, 1.28) | 0.92 | (0.67, 1.26) |
|  | Mild LD | 2.32 | (1.59, 3.40) | 1.80 | (1.26, 2.58) | 1.84 | (1.31, 2.57) | 2.20 | (1.41, 3.43) | 1.59 | (1.06, 2.40) | 1.85 | (1.28, 2.66) | 1.84 | (1.11, 3.06) | 1.41 | (0.90, 2.21) | 1.63 | (1.10, 2.41) |
|  | Diabetes without C.Cx | 1.22 | (0.78, 1.91) | 1.04 | (0.68, 1.60) | 1.03 | (0.68, 1.56) | 1.59 | (0.99, 2.55) | 1.25 | (0.80, 1.96) | 1.23 | (0.80, 1.89) | 1.22 | (0.70, 2.15) | 0.91 | (0.53, 1.54) | 0.96 | (0.59, 1.56) |
|  | Diabetes with C.Cx | 2.10 | (1.28, 3.46) | 2.28 | (1.45, 3.60) | 2.35 | (1.53, 3.63) | 1.76 | (1.03, 2.99) | 1.97 | (1.22, 3.18) | 1.94 | (1.23, 3.06) | 1.56 | (0.87, 2.79) | 2.01 | (1.23, 3.30) | 2.07 | (1.29, 3.32) |
|  | Hemiplegia, paraplegia | 2.76 | (0.83, 9.17) | 3.03 | (1.01, 9.05) | 3.68 | (1.38, 9.78) | 2.10 | (0.49, 8.93) | 2.59 | (0.76, 8.82) | 3.50 | (1.20, 10.24) | 0.97 | (0.13, 7.42) | 1.47 | (0.33, 6.61) | 2.37 | (0.68, 8.22) |
|  | Renal disease | 1.90 | (0.73, 4.90) | 2.03 | (0.79, 5.23) | 2.04 | (0.80, 5.21) | 1.49 | (0.51, 4.30) | 1.60 | (0.56, 4.58) | 1.57 | (0.55, 4.47) | 1.85 | (0.43, 7.96) | 2.81 | (0.83, 9.50) | 2.47 | (0.73, 8.42) |
|  | Moderate or severe LD | 4.62 | (1.94, 10.99) | 6.27 | (2.96, 13.25) | 5.74 | (2.72, 12.13) | 4.87 | (1.86, 12.73) | 6.76 | (3.00, 15.22) | 5.67 | (2.52, 12.76) | 6.85 | (2.33, 20.13) | 9.97 | (4.47, 22.23) | 7.01 | (3.1, 15.86) |

CCI, Charlson Comorbidity Index; HR, hazard ratio; CI, confidence interval; In, inpatient; MI, myocardial infraction; CHF, chronic heart failure; PVD, peripheral vascular disorders; CVD, cardiovascular disease; COPD, chronic obstructive pulmonary disease; PUD, peptic ulcer disease; LD, liver disease; C.Cx, chronic complications

^a)^ Prognostic prediction models were adjusted for sex and all comorbidity conditions except Dementia and AIDS/HIV in Cox proportional hazards model accounting for left truncated and right-censored data.
